# Adequate interval for the monitoring of vital signs during endotracheal intubation

**DOI:** 10.1186/s12871-017-0399-y

**Published:** 2017-08-22

**Authors:** J.Y. Min, H.I. Kim, S.J Park, H. Lim, J.H. Song, H. J. Byon

**Affiliations:** 1 Department of Anesthesiology and Pain Medicine, Severance Hospital, Anesthesia and Pain Research Institute, Yonsei University College of Medicine, 50-1 Yonsei-ro, Seodaemun-gu, Seoul, 120-752 Republic of Korea; 2 Department of Anesthesiology and Pain Medicine, Inha University College of Medicine, Incheon, Republic of Korea

**Keywords:** Vital sign, Monitoring, Intubation, Adequate interval

## Abstract

**Background:**

In the perioperative period, it may be inappropriate to monitor vital signs during endotracheal intubation using the same interval as during a hemodynamically stable period. The aim of the present study was to determine whether it is appropriate to use the same intervals used during the endotracheal intubation and stable periods to monitor vital signs of patients under general anesthesia.

**Methods:**

The mean arterial pressure (MAP) and heart rate (HR) were continuously measured during endotracheal intubation (15 min after intubation) and hemodynamically stable (15 min before skin incision) periods in 24 general anesthesia patients. Data was considered “unrecognized” when continuously measured values were 30% more or less than the monitored value measured at 5- or 2.5-min intervals. The incidence of unrecognized data during endotracheal intubation was compared to that during the hemodynamically stable period.

**Result:**

There were significantly more unrecognized MAP data measured at 5-min intervals during endotracheal intubation than during the hemodynamically stable period (*p* value <0.05). However, there was no difference in the incidence of unrecognized MAP data at 2.5 min intervals or HR data at 5 or 2.5 min intervals between during the endotracheal intubation and hemodynamically stable periods.

**Conclusion:**

A 5-min interval throughout the operation period was not appropriate for monitoring vital signs. Therefore, , a 2.5-min interval is recommended for monitoring the MAP during endotracheal intubation.

## Background

Monitoring vital signs such as mean arterial pressure (MAP), heart rate (HR), respiratory rate, and body temperature during the perioperative period is important for reviewing the hemodynamic status of patients and for planning future treatments [1–3]. Monitored vital signs are also used as basic data for clinical studies and malpractice trials [4–9]. Shorter monitoring intervals allow for vital signs to make greater contributions toward determining patients’ vital sign trends [10, 11]. On the other hand, short intervals demand significant time and effort from the anesthesiologist. According to the American Society of Anesthesiologists (ASA) guidelines and clinical conventions, anesthesiologists tend to monitor vital signs at 5-min intervals during the perioperative period. The 5-min interval for monitoring vital signs can be appropriate when patients are hemodynamically stable. However, when patients are hemodynamically unstable, this interval may be insufficient to accurately assess rapidly changing hemodynamics [10, 12]. Endotracheal intubation is a hemodynamic-stimulant procedure that can cause abrupt changes in the vital signs of patients. However, the interval for monitoring vital signs is usually constant throughout the perioperative period. We believe it would be more appropriate to make the interval change during endotracheal intubation. The aim of this study was to compare the appropriateness of different intervals for monitoring vital signs in the same patients during endotracheal intubation and hemodynamically stable periods.

## Methods

This study was approved by the institutional review board, and informed consent was obtained from subjects. The study was prospectively conducted on 25 adult patients who were 20–60 years old, had either ASA physical status I or II, and were undergoing general anesthesia for elective surgery. Patients with cardiovascular, respiratory, and cerebral disorders were excluded. Patients anticipated to have difficulties with mask ventilation or intubation (cervical spine disease, limited mouth opening and neck extension, pharyngeal pathology, or history of difficult intubation) were also excluded. Fluid was infused intravenously as per the weight of the patients during Non Per Os time to prevent hypovolemia. Midazolam 2–3 mg and Glycopyrrolate 0.5 mg were administered intravenously as premedication. All patients were monitored with an electrocardiogram, pulse oximeter, non-invasive blood pressure, capnography, bispectral index (BIS), and neuromuscular monitoring upon arrival in the operating room. After lidocaine infiltration, a right radial arterial catheter (BD Arterial Cannula, BD Infusion Therapy Systems Inc., Utah, USA) was inserted, and arterial pressure was measured continuously for every patient. Baseline vital signs were defined as the values measured immediately before anesthetic induction. After 1 mg/kg of lidocaine was administered intravenously, anesthesia was induced with propofol (4 mcg/ml of effect-site concentration) and remifentanil (3 ng/ml of effect-site concentration) using target-controlled infusion (Minto model for remifentanil). The effect-site concentrations of propofol (2–5 mcg/ml) and remifentanil (2–5 ng/ml) were adjusted by 1 mcg/ml or 1 ng/ml of the effective-site concentration at 2-min intervals to maintain a target range for BIS (45–60) and MAP within ±30% of the respective baseline values. The effect-site concentrations of propofol and remifentanil were monitored every minute during the endotracheal intubation and stable periods. Three minutes later, rocuronium bromide (0.6 mg/kg) was administered, and neuromuscular blockade was confirmed using a train-of-four (TOF) stimulation count (TOF = 0). The trachea was intubated, and the lungs were mechanically ventilated to maintain normocarbia. During the perioperative period, MAP and HR were maintained according to standard protocols. Rescue medication was administered when MAP and HR could not be maintained by adjusting the effect-site concentration of remifentanil (2–5 ng/ml) or when immediate treatment was needed due to severe abnormal vital signs that indicated a compromised hemodynamic status. If the MAP increased or decreased by more than 30% of the baseline value, a single bolus of nicardipine (300 mcg) or phenylephrine (100 mcg) was given at 3-min intervals up to three times. If the HR increased or decreased by more than 30% of the baseline value despite maintenance of the MAP, a single bolus of esmolol (10 mg) or atropine sulfate (0.5 mg) was given at 3-min intervals up to three times. If MAP and HR were not maintained within 30% of the baseline value despite three administrations of rescue medication, the patient was removed from the present study and received proper treatment such as fluid and inotropics [13–16].

### Collection and analysis of vital signs

We collected and analyzed MAP and HR because respiratory rate and body temperature are relatively constant during anesthesia. The monitor calculated beat-per-beat values for MAP and HR and displayed the mean values over the previous 2 s on the screen. Data was automatically downloaded from the monitor (GE MARQUETTE SOLAR 8000 M, GE MEDICAL SYSTEMS INFORMATION TECHNOLOGIES, Milwaukee, USA) to a personal computer using an RS-232C interface throughout the entire perioperative period at 1-s intervals. With the exception of BIS recorded at 10-s intervals, measured vital signs recorded at 1-s intervals were collected and analyzed. The endotracheal intubation period and hemodynamically stable period were defined as 15 min after endotracheal intubation and 15 min before skin incision, respectively. During both periods, any procedure that influenced the monitoring of vital signs, such as vascular cannulation and foley catheter insertion, was prohibited. For 5-min monitoring intervals, values were monitored at six time points (2.5, 7.5, and 12.5 min after intubation and 12.5, 7.5, and 2.5 min before skin incision). For 2.5-min intervals, values were monitored at 12 time points (1.25, 3.75, 6.25, 8.75, 11.25, and 13.75 min after intubation and 13.75, 11.25, 8.75, 6.25, 3.75, and 1.25 min before skin incision). For example, for the 5-min interval, the measured value at 2.5 min after intubation was considered the monitored value and represented the values measured immediately to 5 min after intubation. Measured values were defined as ‘unrecognized data’ when they were 30% more or less than the monitored value. The incidence of unrecognized data in this study may correspond to unrecognized periods when vital signs were 30% more or less than the monitored vital signs measured at 5- or 2.5-min intervals.

### Statistical analysis

We performed a pilot study involving 10 patients and measured the incidence of unrecognized data for the MAP in each patient. The results of the pilot study showed a mean difference ± SD (%) of 7.1 ± 8.6% in the incidence of unrecognized data for the MAP between the endotracheal intubation and hemodynamically stable periods. The required sample size, calculated with 95% power and α = 0.05 using a paired t-test, was a minimum of 22 participants. Assuming a potential dropout rate of 10%, 25 participants were recruited for the present study. Using 5- or 2.5-min intervals to monitor vital signs, the mean incidence of unrecognized data for MAP and HR were compared using a paired t-test between the endotracheal intubation and hemodynamically stable periods. Bonferroni’s method was used to correct for multiple comparisons. The data is expressed as mean ± SD, as median and interquartile range, and as proportions or counts, based on the data’s characteristics. Comparisons were considered statistically significant when the *p* value was <0.05.

## Results

**Fig. 1:**
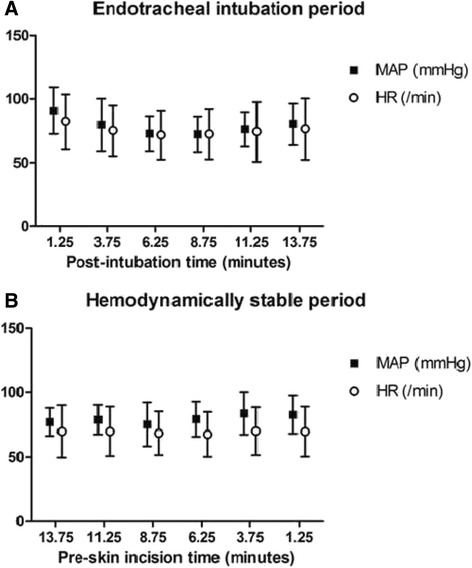
Monitored MAP and HR during the two periods for the 2.5-min-interval group

A total of 25 patients undergoing lumbar spinal fusion were enrolled in this study. The vital signs of 24 patients were analyzed, as one patient was dropped from the study due to uncontrolled blood pressure despite three injections of rescue medicine. The demographic characteristics of patients are shown in Table 1. And the time interval (mean ± SD) between the endotracheal intubation periods and the stable periods was 41.7 ± 15.4 min. Figure 1 shows the vital sign trends during the endotracheal intubation period and the hemodynamically stable period. During endotracheal intubation, the differences between the maximal and minimal MAP and HR (mean ± SD) for each patient were 31.8 ± 20.7 mmHg and 34.9 ± 12.5 per min, respectively. During the hemodynamically stable period, the differences were 17.2 ± 11.3 mmHg and 14.6 ± 15.3 per min, respectively. The incidence of unrecognized data in this study may correspond to unrecognized periods when vital signs were 30% more or less than the monitored vital signs measured at 5- or 2.5-min intervals**.** Significantly high incidence of unrecognized data for the MAP with 5-min intervals was showed during the endotracheal intubation period compared to during the hemodynamically stable period. However, there were no differences between the two periods in the incidence of unrecognized data for the MAP with 2.5-min intervals. (Table 2) The incidence of unrecognized data for the HR was also no different for either time interval. When a 30% difference was observed based on the baseline, patients were injected with appropriate drugs as shown in Table 3.

**Table 1:** Demographic characteristics of patients

Sex (male/female)	13/11
Age (years)	50.8 ± 5.4
Height (cm)	165.3 ± 9.9
Weight (kg)	59.5 ± 13.6

Values are expressed as mean ± SD or number

**Table 2:** Incidences (%) of unrecognized data monitorings of vital signs

Supposed time interval	Vital signs	Endotracheal intubation	Hemodynamically stable period	*p*-value
5 min	MAP	7.9 ± 8.2	2.1 ± 3.8	0.006
	HR	5.1 ± 6.7	2.4 ± 4.2	0.058
2.5 min	MAP	2.5 ± 3.1	1.2 ± 2.2	0.065
	HR	1.9 ± 2.5	1.7 ± 3.2	0.772

MAP and HR mean mean arterial pressure and heart rate respectively

Values are expressed as mean ± SD

**Table 3:** Anaesthetic management and rescue medicine

	Endotracheal intubation	Hemodynamically stable period
Effect-site concentration of propofol (mcg/ml)	4.0 ± 0.5	3.5 ± 0.7
Effect-site concentration of remifentanil (ng/ml)	3.7 ± 0.8	3.1 ± 0.7
Nicardipine injection	5	2
Phenylephrine injection	4	2
Esmolol injection	3	1
Atropine injection	2	2

Values are expressed as mean ± SD or number

## Discussion

During endotracheal intubation, vital signs can change rapidly and widely despite various efforts to reduce the changes by the anesthesiologist. Abrupt changes in vital signs can induce critical cardiovascular complications such as cerebral stroke, angina, and myocardial infarction. During endotracheal intubation, vital sign monitoring is clinically important, particularly in patients susceptible to cardiovascular complications. Although there have been a few studies that evaluated adequate time intervals for monitoring vital signs during the perioperative period, these studies did not focus on endotracheal intubation [10–12]. There has been no research on the appropriate time interval for monitoring vital signs during endotracheal intubation, although the latter induces a hemodynamic response that can cause serious cardiovascular complications. To our knowledge, this is the first study that focuses on finding the appropriate time intervals for monitoring vitals during endotracheal intubation.

In the present study, at 5-min intervals, there was a higher incidence of unrecognized MAP data when monitoring vital signs during the endotracheal intubation period than when monitoring during the hemodynamically stable period. At 2.5-min intervals, there were no differences in the incidence of unrecognized data between the endotracheal intubation and the hemodynamically stable periods. Thus, 2.5-min intervals are recommended for monitoring vital signs in order to reduce the incidence of unrecognized data during endotracheal intubation.

Sympathetic stimulus caused by tracheal intubation can be different based on various factors related to anesthesia and tracheal intubation. To reduce the diversity of patients’ hemodynamic responses to sympathetic stimulation, we used the same anesthetic method, including the anesthetic drug, tracheal intubation technique, and protocol for controlling vital signs. Anesthetics, propofol and remifentanil, were administered using target-controlled infusion to reduce the possibility of inter-individual variability. Remifentanil was given as an opioid analgesic to every patient to decrease excessive increases in MAP and HR. Tracheal intubations were performed by a single experienced anesthesiologist.

The present study focused on the appropriate time interval for monitoring vital signs during endotracheal intubation. During general anesthesia, there can be various types of hemodynamically unstable periods, such as during skin incision, extubation, or massive bleeding. The results of the present study can be applied to other hemodynamically unstable periods during general anesthesia if the trend and range of the vital signs are similar to those of the present study. In this study, the differences between the maximal and minimal MAP and HR (mean ± SD) of each patient were 31.8 ± 20.7 mmHg and 34.9 ± 12.5 per min, respectively, during tracheal intubation. During perioperative periods showing a more severe change in vital signs than in the present study, the time interval for monitoring vital signs should be shorter than 2.5 min. However, monitoring arterial pressure using non-invasive blood pressure at 1-min intervals or using an intra-arterial catheter might cause complications such as an injury to the nerve or an ischemia of the extremes.

The incidence of unrecognized data in this study may correspond to unrecognized periods when vital signs were 30% more or less than the monitored vital signs measured at 5- or 2.5-min intervals. A higher frequency of 30% variations indicates that patients spend more time with abnormal vital signs. In the present study, at 5-min intervals during endotracheal intubation, the MAP of patients was more than 30% different from the monitored vital signs for 71 s. In healthy patients, 71 s of abnormal vital signs is unlikely to induce critical complications. However, in high-risk patients (e.g., the elderly with cardiovascular diseases) rapid changes in vital signs may produce serious complications [1, 17, 18]. Furthermore, in this study, invasive arterial line monitoring was performed to continuously monitor the vital signs. When a 30% difference was observed based on the baseline, patients were injected with appropriate drugs as shown in Table 3 and their vital signs were normalized. However, if hemodynamic changes that require active treatments occur and are not treated, the duration of unrecognized periods may be longer and affect the clinical outcome of patients. At 5-min intervals during hemodynamically stable periods and at 2.5-min intervals during endotracheal intubation periods and stable periods, the incidence of unrecognized data (<25 s) was too low to produce a clinical effect.

In clinical practice, anesthesiologists measure vital signs continuously using intra-arterial catheters when rapid changes in vital signs are predicted or observed during the perioperative period. However, guidelines for establishing the intervals of monitoring vital signs during ordinary perioperative events, such as endotracheal intubation, are still necessary, as endotracheal intubation can cause unpredicted rapid changes in vital signs even in healthy patients. Furthermore, patients may have undiagnosed underlying diseases that may cause a sudden change in vital signs. When rapid changes in vital sign are recognized in a patient without continuous monitoring, shortening the interval for measuring vital signs can facilitate early detection of cardiovascular complications, as per the results of the present study.

The present study had several limitations. First, patients in this study were healthy and young. Applying our results to elderly patients or individuals with cardiovascular disease should be done with caution. Second, MAP and HR during anesthesia are influenced by the anesthetic method, including anesthetic drugs, intubation technique, and the protocol for managing MAP and HR. If a different anesthetic method had been used, vital signs and the incidence of unrecognized data may have been different from those of the present study. Third, the MAP was monitored and managed based on data obtained from continuous monitoring with an intra-arterial catheter. If the MAP were measured using a non-invasive blood pressure monitor with 2.5- or 5-min intervals, the change in vital signs could have been greater than that observed in the present study. This may have resulted in a higher incidence of unrecognized data than found in the present study.

## Conclusion

Our data suggests that vital sign abnormalities may be accurately identified by more frequent measurement during endotracheal intubation. During endotracheal intubation, 5-min intervals may be inappropriate for monitoring vital signs. To reduce the incidence of unrecognized data, 2.5-min intervals are recommended for monitoring the MAP during endotracheal intubation.

## Abbreviations

ASA: American Society of Anesthesiologists
BIS: Bispectral index
HR: Heart rate
MAP: Mean arterial pressure
NIBP: Non-invasive blood pressure
NPO: Non Per Os
TOF: Train-of-four
